# Biomechanical effects of sex and bat size on head-directed blunt strikes

**DOI:** 10.3389/fbioe.2026.1797941

**Published:** 2026-05-12

**Authors:** Luyi Guo, Jin Yang, Yaohan Huang, Jiani Sun, Han Zhang, Shangxiao Li, Weiya Hao

**Affiliations:** 1 Research Center for Sports Psychology and Biomechanics, China Institute of Sport Science, Beijing, China; 2 School of Medicine and Health, Shunde Polytechnic University, Foshan, China

**Keywords:** blunt force injury, forensic biomechanics, kinematics, motion analysis, strikes

## Abstract

**Objective:**

Blunt force injury to the head frequently occurs in violent assaults. It is essential to understand how sex and bat size influence striking performance for forensic reconstruction and injury assessment. This study aims to determine how sex and bat size influence kinematic and spatial parameters during head-directed blunt strikes.

**Methods:**

Thirty-six healthy adults (18 males and 18 females) performed strikes on a PVC dummy’s head using two baseball bats. Three-dimensional motion capture system was used to obtain striking velocity, impact energy, upper-limb joint velocities, and spatial parameters. Two-way repeated-measures ANOVA was applied to evaluate the influence of sex and bat size.

**Results:**

Males exhibited higher striking velocity and energy using both bats, with 41.6% and 30.0% higher in velocity whereas 100.2% and 71.0% higher in energy. Higher striking energy was observed within both males and females when the long bat was used. Males showed significantly higher right shoulder velocity, elbow velocity, and wrist velocity. In addition, both males and females showed higher wrist velocity when using the short bat. Compared with males, females showed significantly larger offender azimuth angle (*θ*) and victim azimuth angle (*φ*). Moreover, *θ* increased for both males and females using the short bat. In addition, larger striking distance (*L*) were consistently observed for both males and females when using the long bat.

**Conclusion:**

Males exhibited higher striking velocity and energy than females. Higher values in energy were observed when the long bat was used for each sex, suggesting a greater likelihood of severe injury. Differences in upper-limb kinematics and spatial parameters further reflected adaptations of sex and bat size. Collectively, these results provide quantitative biomechanical evidence that may support forensic reconstruction and analysis of offender–victim interactions.

## 1 Introduction

Blunt force injuries usually account for a significant proportion of violent crimes and are defined as injuries caused by the application of mechanical external force from a blunt object or surface (Sterzik et al., 2016). In forensic testing and identification practice, death caused by blunt force injury represent one of the most frequently encountered scenarios (Sulaiman et al., 2014). An analysis of 1,233 intentional injury cases with identified causative instruments showed that 57.2% involved blunt force injuries, among which 6.3% were associated with bat-related assaults (Jin et al., 2019).

The head is often targeted in violent attacks as the center of vital physiological functions and a prominent exposed part of the human body (Wang et al., 2023). Blunt head injuries are one of the common modes of death in murder cases (Sulaiman et al., 2014). Ambade and Godbole conducted statistics on 241 homicides in which 80% of the head injuries were caused by blunt force (Ambade and Godbole, 2006).

Previous forensic biomechanical studies have focused on slashing motions with knives (Yuan et al., 2022; Li et al., 2023; Li et al., 2024; Yang et al., 2024), coordinated movements and energy expenditure during attacks with Indigenous Australian weapons (Diamond et al., 2024), and wound morphology induced by pickaxe impacts (Amadasi et al., 2024). In addition, researchers have investigated the effects of rod length and mass (Muggenthaler et al., 2018), as well as different striking techniques (Trinh et al., 2018), on maximal striking velocity. However, the biomechanical characteristics underlying blunt-force head strikes with bat-like weapons remain insufficiently understood, particularly regarding how motion kinematics and spatial characteristics jointly influence striking outcomes.

In forensic investigations, particularly when identifying offenders and the weapons used, it is crucial to understand the mechanical boundaries of both the offender and the weapon. Sex serves as a primary determinant of physical strength and upper-limb kinematics (Kritzer et al., 2024), directly influencing the maximum energy an assailant can deliver. Concurrently, baseball bats are frequently utilized in blunt-force assaults, and variations in bat size (length and mass distribution) significantly alter the moment of inertia (Southard and Groomer, 2003), thereby constraining the swing mechanics and the resulting injury potential.

Therefore, the purpose of this study was to investigate the biomechanical features of the striking using two baseball bats. We hypothesized that (1) sex would have a significant influence on striking velocity, joint velocity, striking energy and spatial parameters, with males have higher values than females and (2) striking with long bat (LB) would generate higher striking velocity, joint velocity striking energy and spatial parameters. The results of this study could offer quantitative evidence that can support criminal investigation, forensic evaluation, and judicial decision in cases involving blunt force injury. Beyond forensic applications, this research provides fundamental insights into the interaction between human and tools during striking actions with maximum effort. Furthermore, these findings contribute valuable baseline data to the broader fields of sports biomechanics by quantifying how the motor system adapts multi-joint kinematics to varying implement inertias and sex.

## 2 Methods

### 2.1 Participants

The sample size was determined through *a priori* power analysis using G*Power (Heinrich-Heine-Universität Düsseldorf, Germany). For a two-way repeated ANOVA (within-between interaction effect), expecting a moderate effect size (Cohen’s f = 0.25) based on prior biomechanical studies of striking motions, and specifying an alpha level of 0.05 and a statistical power of 0.80, the required minimum sample size was calculated to be 34. Consequently, thirty-six participants, consisting of 18 males and 18 females, aged 18–30 years old, were recruited as participants for the striking test to ensure sufficient statistical power while accommodating any potential data attrition (Table 1). All of them were right-handed and in good physical condition, with no history of injury and disease in the past 6 months. Prior to participation in the experiment, all participants signed an informed consent form. This study was approved by the Institutional Review Board.

**TABLE 1:** Basic information of the participants (mean ± standard deviation).

Sex	Age (years)	Height (cm)	Weight (kg)	Arm length (cm)
Male (n = 18)	24.4 ± 1.7	175.2 ± 6.5	77.4 ± 12.1	56.58 ± 3.54
Female (n = 18)	24.6 ± 1.7	163.7 ± 6.1	62.1 ± 10.2	51.66 ± 3.50

### 2.2 Data collection

**FIGURE 1:**
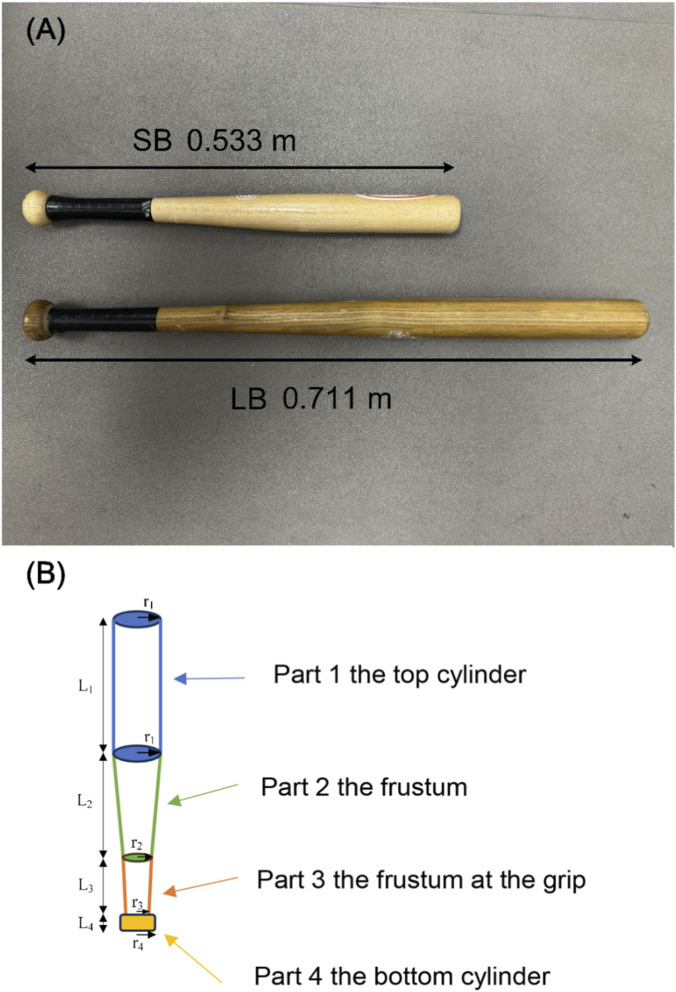
**(A)** The two baseball bats used in the experiment. **(B)** Schematic illustration of the segmentation used for calculating the moment of inertia of the bat. Note: Radii and lengths for the bats. LB: r_1_:2.413 cm, r_2_: 1.651 cm, r_3_: 1.270 cm, r_4_:2.286 cm, L_1_:29.970 cm, L_2_: 27.178 cm, L_3_: 11.94 cm, L_4_: 2.032 cm; SB: r_1_: 2.223 cm, r_2_: 1.429 cm, r_3_: 1.270 cm, r_4_: 2.064 cm, L_1_:21.590 cm, L_2_: 15.558 cm, L_3_: 13.97 cm, L_4_: 2.223 cm.

**FIGURE 2:**
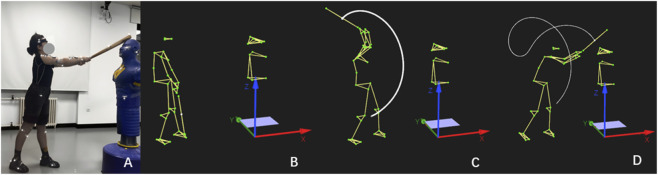
Illustration of the participant’s strike on the dummy using the LB with two hands. **(A)** Moment before contact during an actual videotaped strike; Sketch bat diagram of the striking movement at the moment of start **(B)**, middle **(C)**, and end point **(D)**.

A PVC silicone dummy was used in this study (170 cm length, 65 cm width) as the strike target. During the experiment, the dummy was placed in an upright position and secured on a stable base placed on the floor, as visually depicted in Figure 2. Two baseball bats with different lengths and masses were used as striking implements (TRY&DO SPORTS, Linyi Mushan International Trade Co. Ltd., China). The LB measured 0.711 m in length and weighed 0.792 kg, whereas the short bat (SB) measured 0.533 m in length and weighed 0.448 kg (Figure 1A).

Retro-reflective markers (14 mm), consistent with the Qualisys Sports Marker Set model, were strategically placed. Thirty-five markers were affixed to the participants’ bodies, and 15 markers on the dummy’s head, shoulders, chest, and hips. Bat markers were placed at the distal end, center of mass, and proximal end (Table 2).

**TABLE 2:** Location of the marking point.

Marker	Location	Marker	Location
SGL	On headband, forehead	L/R-FCC	Left/right heel
L/R-HEAD	On headband, just above left/right ear	L/R-FM2	Left/right 2nd toe
SME	Sternum	L/R-FM5	Left/right 5th toe
TV2	Spine, 2nd thoracic vertebra	D.L/R-GON	Dummy’s Left/right gonion
TV12	Spine, 12th thoracic vertebra	D.SGL	Dummy’s sub glabella
SACR	Sacrum (midpoint of the posterior superior iliac spine)	D.BRE	Dummy’s bregma
L/R-SAE	Left/right shoulder	D.EOP	Dummy’s external occipital protuberance
L/R-HLE	Left/right elbow (outside)	D.SME	Dummy’s sternum
L/R-HME	Left/right elbow (inside)	D.MS	Dummy’s mastoid
L/R-RSP	Left/right wrist (thumb side)	D.L/R-ZYG	Dummy’s left/right zygion
L/R-USP	Left/right wrist (pinkie side)	D.L/R-FE	Dummy’s left/right frontotemporale
L/R-HM2	Left/right hand (basis of forefinger)	D.L/R-MAS	Dummy’s left/right mastoidale
L/R-IAS	Left/right pelvis (anterior superior iliac spine)	D.L/R-SAE	Dummy’s left/right shoulder
L/R-PAS	Left/right patella (above knee)	B-ST	Top of the bat
L/R-TTC	Left/right shin	B-C	Center of the mass of the bat
L/R-FLE	Left/right knee	B-SH	Tail of the bat
L/R-FAL	Left/right ankle	​	​

A total of 53 markers were affixed on the participants (35), dummy (15), and bat (3).

Participants were instructed to strike the frontal region of the dummy’s head using bats with maximum effort after a warm-up period. No much strict constraints were imposed on striking process to ensure the strikes simulated a real assault rather than a motor learning task. Each participant was required to strike three times with both LB and SB while holding the bat with two hands. The testing order for LB and SB was determined using a Latin Square design. Each trial began and ended with the bat naturally resting at the participant’s side. Participants were permitted to adjust their distance and angle to the dummy to maximize strike power. The strike trial with the highest bat center-of-mass velocity immediately prior to contact was selected for subsequent analysis (Figure 2). Kinematic data were collected using an 8-lens Qualisys 3D motion capture system (Oqus700, Qualisys Track Manager, Sweden) at a sampling rate of 250 Hz, and processed using the Qualisys Track Manager software.

### 2.3 Data processing

Kinematic data were filtered using a 4th-order Butterworth low-pass filter with a cut-off frequency of 25 Hz (Li et al., 2024). The striking process was defined as initiating from a resting posture, specifically beginning at the frame where the bat’s center-of-mass velocity exceeded zero and continued to increase without returning to baseline. The process ended at the exact moment of impact, identified as the frame when the velocity of the reflective marker on the dummy’s sub-glabella exceeded zero. Striking velocity is defined as the velocity of the center of mass of the bat at the contacting moment. The striking energy generated at the moment the baseball bat touched the dummy is the sum of the translational and rotational kinetic energy (Equation 1).

$$E=\frac{1}{2}mv_{c}^{2}+\frac{1}{2}J\omega^{2}$$ (1)

Where m is the mass of the bat, v_c_ is the velocity of the center of mass of the bat, J is the moment of the inertia of the bat and ω is the angular velocity of the center of mass of the bat.

The calculation for the moment of inertia was based on theory of mechanics and as follows: The baseball bat was divided into four parts, the top cylinder, the frustum, the frustum at the grip and the bottom cylinder (Figure 1B). Both baseball bats were assumed to have a uniform density; therefore, the mass of each segment was determined by multiplying its calculated volume by this density (LB: 777.45 kg/m^3^, SB: 734.09 kg/m^3^). The moment of inertia for each part of a bat was calculated separately, then the parallel axis theorem was applied to calculate the moment of inertia for each part rotating around an axis through the center of mass perpendicular to the long axis of the bat (transverse axis). The sum of these is considered the moment of inertia of the baseball bat (Equation 2).

$$J=\sum_{i=1}^{4}\left(J_{i}+m_{i}\cdot d_{i}^{2}\right)$$ (2)

Where *J*_*i*_ is the moment of inertia of the *i*-th segment about its own center of mass, *m*_*i*_ is the mass of the *i*-th segment, and *d*_*i*_ is the perpendicular distance from the center of mass of the *i*-th segment to the transverse axis of the baseball bat.

All calculations were performed in MATLAB R2024a. The moment of inertia of LB and SB was calculated to be 0.0294 kg·m^2^ and 0.0097 kg·m^2^ respectively.

Joint velocity (shoulder, elbow, wrist) was defined as the maximal velocity for the joint center during the striking process.

**FIGURE 3:**
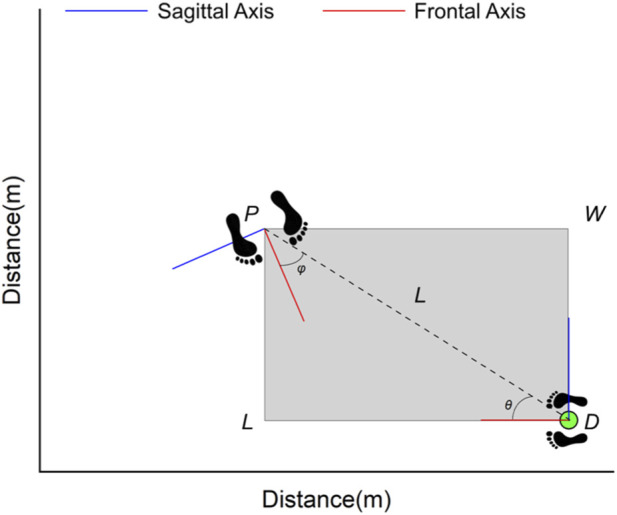
Schematic diagram of spatial parameters between participants and dummy during the contact moment, including offender azimuth angle (*θ*), victim azimuth angle (*φ*), striking distance (*L*), and occupancy area (*S*). The blue and red solid lines are the sagittal and frontal axes of the participants and dummy. The larger footprints marked with point *P* represent the positions of participants when striking the dummy, while the small footprint with a green circle and point D represent the position of the dummy. The grey-shaded area enclosed by the WDLP represents the occupancy area.

The spatial indicators were determined at the moment of bat-dummy contact, and were projected onto the global horizontal plane. The parameters are defined as follows (Yang et al., 2024) (Figure 3):

Offender azimuth angle (*θ*): the intersection of the dummy’s sagittal axis and the vector connecting the centers of the participant and the dummy.

Victim azimuth angle (*φ*): the angular deviation between the participant’s sagittal axis and the linear path connecting their center of mass to the dummy’s position.

Striking distance (*L*): the linear separation between the dummy’s center and the participant’s pelvic center. To normalize for anthropometric variations, a relative distance (*L%*) was computed as the ratio of *L* to the participant’s body height.

Occupancy area (S): the spatial encompassed by the participant and the dummy at the moment of contact, quantifying the essential floor space required to execute the strike.

### 2.4 Statistical analysis

The data were tested for normal distribution using the Shapiro-Wilk test. A two-way repeated ANOVA was conducted to examine if sex (male/female, between groups) and bat size (long/short within group) significantly influence the striking velocity, energy, joint velocity, *θ*, *φ*, *L*, *L*% and *S*. If a significant interaction effect is observed, a simple effect analysis would be conducted to further interpret the influence of sex and bat size on the metrics with a Bonferroni correction. Data were expressed as mean ± standard deviation, and all statistical analyses were carried out in SPSS 27.0.1 (IBM, United States) at a test level of α = 0.05.

## 3 Results

**FIGURE 4:**
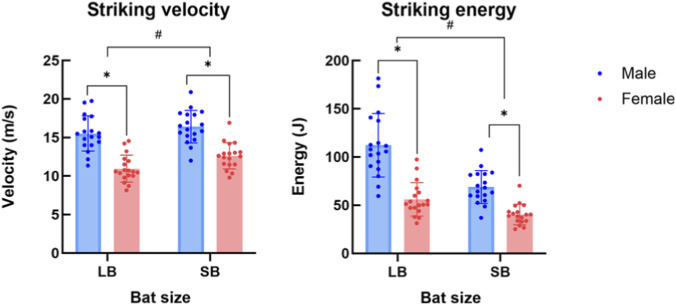
Comparison of striking velocity and energy between sexes and across bat sizes. ^*^indicates that *P* < 0.05 between sexes; ^#^indicates that *P* < 0.05 between bat sizes.

The results of the two-way repeated-measures analysis of variance revealed a significant interaction effect between sex and bat size on both striking velocity and striking energy (*P* < 0.05). Simple effects analysis indicated that both males and females exhibited higher striking velocity when using SB compared with LB, and males demonstrated higher striking velocities than females when using both LB and SB (*P* < 0.05). Moreover, the difference in striking velocity between bat types was greater in females than in males. Regarding striking energy, both males and females generated higher striking energy when using the heavier LB compared with the lighter SB unsurprisingly, and males produced greater striking energy than females with both bat types (*P* < 0.05). In contrast to striking velocity, the difference in striking energy between bat types was greater in males than in females (Figure 4; Table 3).

**TABLE 3:** Comparison of striking velocity, energy and joint velocities between sexes and across bat sizes.

Parameter	Male		Female		*P* value		
	LB	SB	LB	SB	Sex	Bat size	Interaction effect
Striking velocity (m/s)	15.51 ± 2.29	16.40 ± 2.10	10.95 ± 1.74	12.62 ± 1.70	<0.001^*^	<0.001^#^	0.039^&^
Striking energy(J)	112.17 ± 32.96	68.91 ± 16.97	56.02 ± 17.34	40.30 ± 10.71	<0.001^*^	<0.001^#^	<0.001^&^
Right shoulder velocity (m/s)	2.70 ± 0.88	2.77 ± 0.75	2.00 ± 0.63	2.12 ± 0.54	0.005^*^	0.334	0.791
Left shoulder velocity (m/s)	1.63 ± 0.53	1.68 ± 0.68	1.31 ± 0.48	1.29 ± 0.56	0.890	0.058	0.591
Right elbow velocity (m/s)	4.45 ± 1.58	4.62 ± 1.43	3.19 ± 1.03	3.57 ± 1.08	0.008^*^	0.064	0.446
Left elbow velocity (m/s)	3.77 ± 0.92	3.79 ± 0.98	2.94 ± 0.69	2.93 ± 0.57	<0.001^*^	0.968	0.926
Right wrist velocity (m/s)	5.78 ± 1.04	6.67 ± 1.02	3.93 ± 0.91	5.04 ± 1.05	<0.001^*^	<0.001^#^	0.493
Left wrist velocity (m/s)	5.78 ± 0.84	6.05 ± 0.63	3.83 ± 0.78	4.66 ± 0.89	<0.001^*^	<0.001^#^	0.022^&^

* indicates that *P* < 0.05 between sexes.

^#^ indicates that *P* < 0.05 between bat sizes.

^&^indicates that *P* < 0.05 in the interaction effect.

**FIGURE 5:**
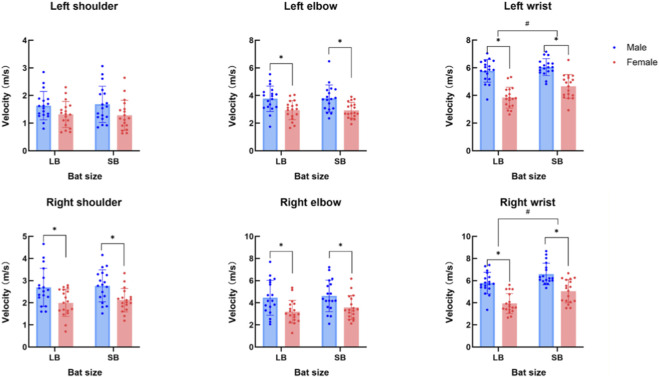
Comparison of joint velocity between sexes and across bat sizes. ^*^indicates that *P* < 0.05 between sexes; ^#^indicates that *P* < 0.05 between bat sizes.

An interaction effect between sex and bat size was observed for the left wrist velocity (*P* < 0.05). Simple effect analysis further revealed that males exhibited higher left wrist velocity than females when using both bats. In addition, both males and females generated greater right wrist velocity when using SB compared with LB. There was a main effect of sex for right shoulder velocity, right elbow velocity, left elbow velocity and right wrist velocity (*P* < 0.05), with higher values shown in males compared with females. No significant main effect of sex was observed for left shoulder velocity (*P* > 0.05). A significant main effect of bat size was detected for right wrist velocity (*P* < 0.05), with higher values shown when using SB. No main effect of bat size was observed for right shoulder velocity, left shoulder velocity, right elbow velocity and left elbow velocity (*P* > 0.05) (Figure 5; Table 3).

The interaction effect between sex and bat size was not significant for *θ*, *φ*, *L*, *L%* and *S* (*P* > 0.05). There was a main effect of sex for *θ*, *φ*, *L%* (*P* < 0.05). Females exhibited larger *θ* and *φ* with both bats. No significant main effect of sex was found for *L* and *S* (*P* > 0.05). A significant main effect of bat size was found for *θ* and *L* (*P* < 0.05), and no significant main effect of bat size was found in *φ*, *L%* and *S* (*P* > 0.05). Both males and females exhibited larger *θ* with SB than LB. Conversely, greater *L* were consistently observed in trials performed with the LB than with the SB, regardless of sex (Table 4).

**TABLE 4:** Comparison of offender-victim spatial parameters between sexes and across bat sizes.

Parameter	Male (n = 18)		Female (n = 18)		*P* value		
	Long	Short	Long	Short	Sex	Bat size	Interaction effect
*θ* (deg)	12.23 ± 7.74	16.17 ± 7.67	16.73 ± 8.13	20.36 ± 12.59	0.013*	0.040^#^	0.935
*φ* (deg)	31.01 ± 13.72	28.03 ± 12.78	38.51 ± 16.39	39.87 ± 15.71	0.019*	0.771	0.338
*L* (m)	0.96 ± 0.12	0.81 ± 0.09	0.90 ± 0.13	0.77 ± 0.07	0.130	<0.001^#^	0.393
*L*%	0.55 ± 0.06	0.46 ± 0.05	0.55 ± 0.07	0.47 ± 0.03	<0.001*	0.747	0.574
*S* (m^2^)	0.18 ± 0.11	0.17 ± 0.09	0.22 ± 0.11	0.18 ± 0.10	0.377	0.914	0.483

*θ*: offender azimuth angle; *φ*: victim azimuth angle. *L*: striking distance; *L%*: relative striking distance; *S*: occupancy area.

* indicates that *P* < 0.05 between sexes.

^#^ indicates that *P* < 0.05 between bat sizes.

## 4 Discussion

Results from this study could offer important information for forensic investigations and court trials. The results completely support our first hypothesis that sex significantly influences striking velocity, joint velocity, and striking energy, with males generating higher values than females. Moreover, our second hypothesis was also partially proved. Both males and females had higher values in striking energy when using LB compared to SB.

It was shown that male participants achieved average striking velocities of 15.51 m/s with LB and 16.40 m/s with SB, whereas females recorded velocities of 10.95 m/s and 12.62 m/s under the same conditions. These striking velocities produced impact energies of 112.17 J and 56.02 J for males and females with LB, and 68.91 J and 40.30 J with SB. Although this study did not directly evaluate injurious outcomes, these values can be contextualized using established injury metrics. Previous studies indicate that the kinetic energy required to initiate a cranial fracture from blunt trauma typically ranges from 14 to 68 J (Yoganandan et al., 1995). Because our recorded energies consistently meet or exceed these tolerance limits, the strikes demonstrate a high potential to inflict severe cranial fractures. The magnitudes of these outputs are also broadly comparable to those reported in other controlled offensive-action studies. For example, Li et al. documented slashing velocities of approximately 10–13 m/s and associated energies of 25–48 J in Chinese kitchen-knife attacks on a dummy (Li et al., 2023). Lower energy level was observed in knife attacks, which highlight the dominant role of implement mass and moment of inertia in blunt-force strikes. In contrast to slashing motions, where tissue damage is primarily governed by edge sharpness and cutting mechanics, blunt strikes rely heavily on kinetic energy transfer, which scales with both velocity and inertial properties of the weapon. This distinction becomes more evident when comparing our results with those of Sprenger et al., which reported bat-striking velocities in the range of 7–12 m/s and energies spanning 67–311 J depending on implement mass and moment of inertia (Sprenger et al., 2016). The broad energy range observed in their study underscores the sensitivity of blunt-force impact severity to weapon mass distribution and rotational inertia.

The result of our study indicated that males generated higher striking velocity and energy than females with both bats, with males achieving up to 41.6% higher striking velocity and nearly twice the energy output when using LB. When using SB, males demonstrated 30.0% greater striking velocity and 71.0% higher energy output relative to females. This finding is consistent with previous research and can be attributed to the fact that males generally possess greater muscle mass and upper-limb strength than females (Muggenthaler et al., 2018). However, we must acknowledge that quantitative data regarding the participants’ physical strength were not collected in the present study. Future research should explore the potential correlation between individual strength levels and the resulting striking velocity and energy, which would further assist in offender profiling in forensic investigations.

Height and weight were introduced as covariates in the analysis to determine whether these disparities were purely functions of sex and bat size. After incorporating these anthropometric variables, the main effect of sex on both striking velocity and energy remained statistically significant (Table 5). This suggests that these differences are likely associated with sex-related physiological characteristic that likely reflects differences in upper-body power generation, rather than merely a byproduct of being taller and heavier. In contrast, the main effect of bat size, as well as the interaction between sex and bat size on striking velocity, became non-significant after controlling for height and weight. This reveals that the kinematic differences originally observed between the two bats are fundamentally driven by the body dimensions of the participants. Specifically, height and weight of an individual dictate their biomechanical capacity to overcome the greater moment of inertia of the longer bat, thereby neutralizing the independent effect of weapon size and the sex interaction when body size is statistically equalized.

**TABLE 5:** Comparison of striking velocity and striking energy between sexes and bat sizes, incorporating height and weight as covariates.

Parameter	*P* value				
	Sex	Stick type	Interaction effect	Height (Covariate)	Weight (Covariate)
Striking velocity (m/s)	<0.001*	0.298	0.710	0.369	0.092
Energy(J)	<0.001*	0.408	0.002^&^	0.229	0.053

* indicates that *P* < 0.05 between sexes.

^&^indicates that *P* < 0.05 in the interaction effect.

Consistent with the whole-body striking performance observed in this study, the joint velocity results demonstrated significant differences between sexes across multiple segments. Male participants exhibited higher velocities in both proximal and distal joints, with particularly pronounced differences at the wrist and elbow. For example, males achieved right wrist velocities of 5.78 ± 1.04 m/s with LB and 6.67 ± 1.02 m/s with SB, whereas females reached 3.93 ± 0.91 m/s and 5.04 ± 1.05 m/s under the same conditions. Similar patterns were present at the elbow and shoulder. These findings correspond with previous descriptions of sequential segmental motion in striking tasks such as baseball batting, in which elevated distal segment velocities are strongly associated with increased end-point velocity and striking performance (Putnam, 1993). The comparison between LB and SB conditions further highlights the functional differentiation of joint contributions in relation to bat size. Although shoulder and elbow joint velocities did not differ significantly between bat sizes, both wrists exhibited marked increases when participants used SB. Moreover, the elevated wrist velocities recorded when using SB correspond with the higher striking velocities observed in our study, suggesting that lower inertial resistance facilitates more efficient distal-segment movement during the forward swing.

In addition to kinematic performance, the spatial parameters quantified in this study provide important insight into how participants adjusted their body position and striking strategy to accommodate different bat sizes and individual characteristics. Female participants consistently exhibited larger *θ* and *φ* values than males with both bats, reflecting a greater angular deviation of the striking trajectory relative to the target. In contrast, no significant sex differences were found for absolute *L* and *S*. This pattern suggests that while males and females maintained comparable overall distances and contact regions, females tended to adopt more oblique or laterally oriented striking approaches. Such spatial adaptations are a little different with a previous research for slashing using knives with one hand, in which males exhibited significantly higher values in *L* and *S* (Yang et al., 2024). This discrepancy is likely attributed to fundamental differences in weapon size and grip style. Slashing with a knife in one hand may exhibit greater movement variability, whereas striking with a bat in two hands constrains the freedom of movement strategy.

The pronounced influence of bat size on striking distance also suggests that implement geometry dominated characteristics related to reach, potentially masking more subtle differences of sex. Both male and female participants demonstrated larger *θ* values when using SB compared with LB, indicating that SB required greater angular adjustment to achieve an effective striking trajectory. Conversely, significantly greater striking distances were observed when participants used LB, reflecting the extended effective reach afforded by the longer implement. These findings highlight that bat geometry primarily constrained the spatial configuration of the strike by altering approach distance and angular alignment, rather than influencing relative distance normalization.

The integration of kinematic and spatial data in this study may provide assistance for forensic scene reconstruction in cases involving blunt-force head strikes. Quantitative parameters such as striking velocity, energy, and azimuth angles can serve as objective indicators for estimating the potential severity of injury, the likely striking posture, and even the sex-related characteristics of a criminal. For instance, the higher striking energy produced by male participants when using the LB may correspond to more severe traumatic brain injury (TBI), as previous research has demonstrated that there is a linear relationship between the severity of TBI and collision energy (Li et al., 2021). Meanwhile, the higher striking velocity associated with the SB may correspond to repetitive or multi-directional impacts. These distinct characteristics can assist in guiding investigation when combined with witness testimony, scene evidence, or suspect anthropometrics.

Beyond forensic reconstruction, these kinematic parameters define the essential boundary conditions for evaluating specific craniocerebral injury mechanisms. In particular, the exacerbation of injury risk at higher striking velocities is consistent with the rate-dependent viscoelastic behavior of cranial bone. As demonstrated by Yoganandan et al. , the human skull exhibits a significant stiffening that depends on the rate of loading during dynamic impacts (Yoganandan et al., 1995). Due to this transition toward a more brittle mode of failure, less absorbed energy may be required to initiate fracture under high-velocity conditions. Consistent with this rate-sensitive mechanical response, a recent finite element simulation study utilizing the experimental velocities demonstrated that increasing strike velocity causes the von Mises stress distribution on the skull to transition from localized concentration to multipolar dispersion. Furthermore, specific injury thresholds were identified, revealing that intracranial pressure exceeds the severe injury limit (235 kPa) when striking velocities surpass 12 m/s for the long bat and 14 m/s for the short bat, which equates to roughly 55 J of kinetic energy, reaching a severely life-threatening level. Thus, the kinematic data obtained in this study not only aid in identifying the offender but also provide a scientific basis for assessing the potential lethality of the trauma (Zhang et al., 2026). In our current study, the average striking velocities generated by male participants (LB: 15.51 m/s; SB: 16.40 m/s) exceeded these critical thresholds, while the velocities generated by female participants (LB: 10.95 m/s; SB: 12.62 m/s) approached them. In terms of striking energy, the majority of our participants reached or exceeded this critical level, with only females using the SB producing slightly lower values. This indicates a high probability of severe or fatal brain trauma in such strikes.

However, several limitations should be acknowledged. First, the posture of the dummy was fixed, whereas in real scenarios, a striking target may be either conscious or unconscious. A conscious victim may attempt to evade the strike, while an unconscious victim may exhibit postural deviations relative to the standardized dummy setup, both of which would influence the kinematic characteristics of the strike and the associated spatial parameters. Consequently, it is strictly probabilistic to use these kinematic data obtained from laboratory settings to infer the sex or identity of an offender. In actual forensic investigations, a multivariate assessment that comprehensively considers the height, segment sizes and physical strength of potential suspects is essential, rather than relying on isolated strike data.

Additionally, our study focused solely on the striking process and did not address injurious outcomes. Future research could instrument the dummy to investigate the relationships between impact parameters, such as impact force, and resulting injuries like skull fractures or traumatic brain injuries. Furthermore, it is important to acknowledge that the target utilized in this study was a PVC silicone dummy. While this setup provided a highly normalized and repeatable target for evaluating the kinematic capabilities of the offenders, there are significant structural and material differences between PVC silicone and human biological tissues. The dummy cannot perfectly replicate the complex viscoelastic properties, energy absorption, and fracture mechanics of a real human head. Consequently, the striking energy calculated in this study primarily represents the kinetic energy delivered by the bat immediately prior to contact, rather than the exact mechanical energy absorbed by and deforming biological tissues upon impact. Finally, the head is structurally complex, and strikes to different anatomical regions may yield substantially different injury outcomes even under similar striking conditions. Therefore, future studies could refine the target area and further examine regionally specific injury susceptibility to improve the forensic interpretation of blunt-force head injury.

## 5 Conclusion

Males exhibited much higher striking velocity and energy than females, suggesting a greater potential for severe injury. Crucially, the statistical adjustment for height and weight confirmed that this superior kinetic output is an inherent sex-specific trait, independent of overall body size. Although the SB allowed for faster striking velocity, the LB consistently produced greater striking energy, highlighting the dominance of implement mass and moment of inertia in energy transfer. Upper-limb velocities mirrored these trends, with higher joint velocities observed in males and during SB trials.

Both sex and bat size affected the spatial strategies adopted by the strikers. Females tended to employ more oblique striking trajectories. Furthermore, in forensic cases, the length of the attacking instrument should be considered, as when using a longer bat, greater striking distances were observed for both males and females. Overall, these findings provide biomechanical evidence that may assist forensic experts in crime scene reconstruction and offender–victim interaction analysis.

## Data Availability

The raw data supporting the conclusions of this article will be made available by the authors, without undue reservation.
